# Mandibular Canal Widening and Bell's Palsy: Sequelae of Perineural Invasion in Oral Cancer

**DOI:** 10.1155/2016/3010934

**Published:** 2016-11-29

**Authors:** Gopinath Thilak Parepady Sundar, Vishwanath Sherigar, Sameep S. Shetty, Shree Satya, Sourabh M. Gohil

**Affiliations:** ^1^Department of Oral and Maxillofacial Surgery, A.B. Shetty Memorial Institute of Dental Sciences, Mangalore, India; ^2^K.S. Hegde Charitable Hospital, Mangalore, India; ^3^Department of Oral and Maxillofacial Surgery, Manipal College of Dental Sciences, Manipal University, Manipal, India

## Abstract

Perineural invasion is an underrecognized route of metastatic spread along the nerve bundles within the nerve sheath into the surrounding tissues. It hinders the ability to establish local control as tumour cells can traverse along nerve tracts well beyond the extent of any local invasion rendering them inoperable and unresectable. Perineural invasion is a marker of poor prognosis. Oral submucous fibrosis with oral cancer constitutes a clinicopathologically distinct disease. Our case highlights an enigmatic presentation of oral submucous fibrosis and its coexistence with oral cancer presenting with unusual neurological disturbance of the inferior alveolar nerve and facial nerve and diffuse widening of the mandibular canal. The objective of this case report is to enumerate the significance of perineural invasion in determining the course of the disease and necessitate the need for future studies that can shed light on molecular mediators and pathogenesis of perineural spread.

## 1. Introduction

The mandibular canal widening is an unusual presentation related only to a few pathologic conditions affecting the lower jaw. Loss of cortical bone surrounding the mandibular canal appears as wide radiolucency on a radiograph, inferred as canal widening [1]. Generalized widening of the mandibular canal may indicate pathologies of neural tissue origin or those that secondarily invade the neural tissue [2, 3].

Perineural invasion (PNI) is considered as a distinct third mode of tumour metastasis for oral squamous cell carcinoma (OSCC) together with lymphatic and blood vessel invasion [4]. It can be detected by the histological presence of tumour cells inside the neural space or by imaging techniques [5]. The trigeminal and facial nerves are commonly infiltrated by the invading tumour cells, resulting in sensory as well as motor function disturbances in the head and neck region [6]. Ambiguous symptoms unrelated to primary site of origin often obfuscate the diagnosis. Clinicians need to be cognizant of multiple hidden causes of paraesthesia in the head and neck region that can have a local or a distant origin.

## 2. Case Report

**Figure 1:**
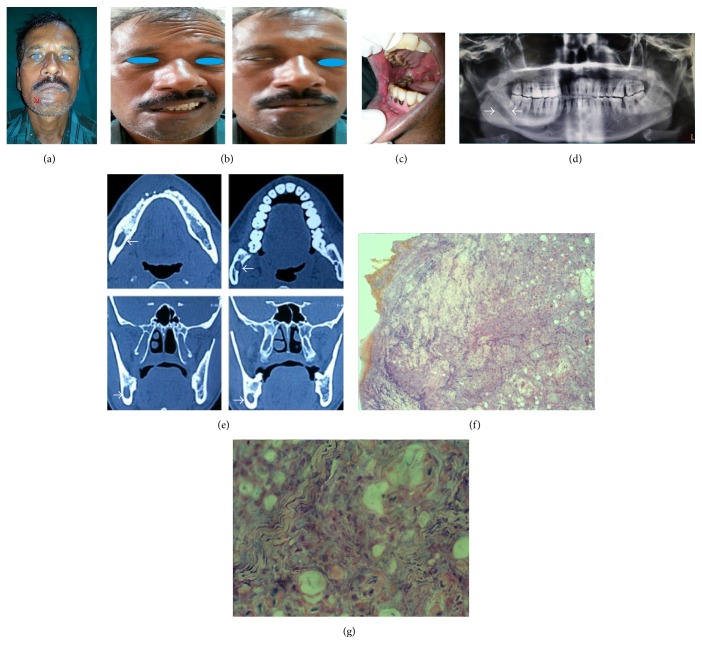
(a) Extraoral draining sinus. (b) Bells sign positive with absence of wrinkles on the affected side of the forehead. (c) Erosive ulcer extending from the corner of the mouth to retromolar trigone. (d) Diffuse widening of the mandibular canal extending from the mandibular foramen to the mental foramen. (e) Axial CT scan showing widening of the mandibular canal. (f) 10x view showing extensive PNI with the presence of tumour cells in the form of islands approximating the neural tissue. (g) 40x view showing intraneural tissue intermixed with tumour islands.

A 48-year-old man with a swelling on the right side of his face below the lower lip reported to our Department of Oral and Maxillofacial Surgery. He presented with a six-month history of nonhealing ulcer in the right side of the buccal mucosa with an extraoral draining sinus and dysphagia for one month (Figure 1(a)).

In addition, he also presented with a two-month history of inability to close his right eye and deviation of the corner of the mouth to the left side followed by numbness in the lower lip and chin region. General health status of the patient and blood and urine analyses were unremarkable. Extraoral examination confirmed the classical signs of lower motor neuron type facial nerve palsy which included absence of wrinkles on the forehead, lagophthalmos of the right eye positive bells sign, flattening of the nasolabial fold [7], and deviation of the angle of the mouth on smile to the left side (Figure 1(b)). Paraesthesia of lower lip and chin suggested infiltration of the inferior alveolar nerve.

Intraoral examination revealed an erosive lesion approximately 6 × 2 cm in size, extending from the angle of the mouth on the right side anteriorly up to the retromolar trigone posteriorly. The floor was covered with a pseudomembranous slough, with rolled edges and erythematous margins. On palpation, there was induration, tenderness, and the presence of fibrous bands (Figure 1(c)). Soft, discrete, mobile submental lymph nodes and bilateral palpable soft submandibular lymph nodes were noted.

The panoramic radiograph revealed the presence of generalized bone loss with diffuse uniform enlargement of the mandibular canal, starting from the mandibular foramen to the mental foramen (Figure 1(d)). Spherical radiolucency and enlargement in the (R) mandibular canal were appreciated in multislice CT [8]. No breach in the cortical plates was seen (Figure 1(e)).

Incisional biopsy of the right buccal mucosa confirmed the clinical diagnosis of squamous cell carcinoma of right gingival-buccal sulcus. Histopathology sections revealed pleomorphic tumour cells with individual cell keratinization and dense peritumoural inflammatory response. Representative histological section demonstrating PNI (Modified Liebig Type A Classification) and infiltration of the epineurium was also seen [9, 10] (Figures 1(f) and 1(g))

The treatment plan included full-thickness, wide local excision of buccal mucosa, segmental mandibulectomy, and modified radical neck dissection preserving internal jugular vein, spinal accessory nerve, followed by reconstruction with the free fibula graft using reconstruction plate. The resected specimen showed well differentiated squamous cell carcinoma with margins and nodes free of tumour. Adjuvant radiotherapy was planned considering the PNI.

## 3. Discussion

Perineural invasion is well defined when at least 33% of the circumference of the nerve is surrounded by tumour cells. The biologic mechanism of PNI pathogenesis remains elusive with postulations that it relates to reciprocal signaling interactions and the intrinsic capacity of tumour cells to retort to signals within the peripheral nerve and promote invasion [11].

PNI is “a tropism of tumour cells for nerve bundles in the surrounding tissues” [4]. It is often linked with an aggressive behaviour, poor prognosis, recurrence, and higher likelihood of regional and distant occult micro and macro metastasis [12]. PNI has also been linked to stimulation of axogenesis that can lead to increased nerve density in and around neurotropic malignancies and further exacerbate tumour progression. PNI detection criteria include histopathological examination of the neural invasion, radiographic examination for osseous canal or foramen widening, and sensory complaints along the nerve distribution [13].

PNI is an important predictor for outcome of patients with SCC of the oral cavity and oropharynx. It is significantly associated with tumour differentiation, lymph node metastasis, depth of invasion, locoregional recurrence, and distant metastasis. Rahima et al. [14] concluded in their study that the 5-year disease-specific survival for patients with and without PNI was 56.6% and 94.6%, respectively (*P* < .0001).

The centrifugal and centripetal propagation of squamous cell carcinoma along the perineural space can demonstrate intracranial spread and jeopardise the available treatment options.

PNI in adenoid cystic carcinoma is well recognized; however, it should also be corroborated in oral squamous cell carcinoma specimens that have of late demonstrated PNI. The presence of PNI can connote a late stage disease and is a hallmark of subclinical invasion that can necessitate the need for aggressive resection, coincident management of neck lymph nodes, and the addition of adjuvant therapy. Targeted drug therapy for PNI in oral cancer is still at its infancy and can be a boon in the years to come. Patients with oral cancer often undergo adjuvant radiation for pathologically high risk features including positive nodal disease, extracapsular spread, and positive tumour margins. Excluding these high risk features, PNI is an independent risk factor necessitating the need for adjuvant radiation [15].

Widening of the mandibular canal is a classical sign of neurofibromatosis but it has been unusual so far in oral cancer. Intraosseous schwannomas may sometimes cause distension of the inferior alveolar canal; they typically produce well-defined unilocular radiolucency mimicking an odontogenic cyst or a tumour. The other causes of mandibular canal widening like perineuroma, multiple endocrine neoplasia syndrome type 2b, vascular leiomyoma, arteriovenous malformation, and traumatic neuroma have been reported in the literature [8].

The persistent paraesthesia of lower lip and chin region is a warning sign of a tumour or a malignancy compressing or invading the nerve.

Facial nerve and the trigeminal nerve are assumed to form a synapse at three strategic locations:

1. Sphenopalatine ganglion
2. Junction of the chorda tympani and the lingual nerve
3. Parotid gland along the auriculotemporal branch of the mandibular nerve

Koivisto et al. (2016) [16] advocated that these synaptic points could provide a viable route for interneural spread of carcinoma from one nerve to another. The facial nerve palsy observed in our patient could be attributed to facial nerve involvement by the tumour cells that might have spread via the mandibular nerve.

The prognostic utility of PNI with respect to the diameter of the nerves is debated with conflicting results being reported from different series of cases. It has been postulated that the stroma of the perineural sheath promotes tumour growth, with the perineural space as a conduit for tumour growth. In view of this large diameter, nerves closer to the surgical margins may be associated with recurrence [17]. Contrary to this, Fagan et al. [18] in their study opined that PNI of small nerves is associated with an increased risk of local recurrence and cervical metastasis and is independent of extracapsular spread, a significant prognostic factor. A review by Woolgar cites OSCC demonstrating that PNI are all related to reduced survival rates and a significant risk of locoregional recurrence irrespective of the diameter of the nerves [19].

## 4. Conclusion

Oral squamous cell carcinoma often presents with a nonhealing exophytic/endophytic ulcer fixed to the underlying skin or the mucosa. This case differs from its usual presentation by its aggressive nature, multiple neural invasion, synchronization of submucous fibrosis and oral cancer, and diffuse widening of the mandibular canal. PNI is an independent risk factor for occult metastasis along with depth of invasion, size of primary tumour, differentiation, and immunosuppression. Further studies are warranted to elucidate its molecular biology of pathogenesis, histopathological pattern of PNI, and its significance in the prognosis. With a better understanding of the mechanisms involved, we can progress to develop therapeutic agents that can target this form of intriguing tumour spread.
